# Strangulated Hernia

**Published:** 1835

**Authors:** C. R. Cooper

**Affiliations:** Assistant Editor; Cincinnati


### V.—Strangulated Hernia.

*Case of an Inguinal Hernia which had existed two years without inconvenience, but finally becoming strangulated, an operation was demanded.* It was performed by Wm. Mount, M. D., of Cummingsville, O. Reported by C. R. Cooper, M. D., Assistant Editor.

On the 2d of May last, Dr. Mount and I were called in consultation with Dr. Bailey, (eight miles from the city,) in the case of Mrs. A———, aged about 30. The report she gave of herself was, that in consequence of excessive fatigue from walking and over exertion, the day previous, her bowel had protruded, and that since then she had been suffering much pain in the part, accompanied by vomiting. She carefully concealed the fact that she had been ruptured two years before, and that the tumor had always remained, though somewhat lessened in size. At the time we saw her she was laboring under some pain, and tenderness of the entire abdominal region, more particularly across the umbilicus—did not complain of very acute pain in the tumor, except upon pressure. The taxis was immediately resorted to, but without any advantage,—as auxiliaries to it, free blood-letting, nauseants, the tobacco-poultice to the part, (strongly recommended in the Journal of Professor Jameson, of Baltimore, now Professor of Surgery in the medical department of Cincinnati College) were prescribed, yet still without the slightest benefit. It was not till every effort failed, every hope of relief (by the means ordinarily used) fled, and the strength of the patient declining, that it was determined to resort to the knife.

The situation of the patient was then, as before, fully explained to her; the dangers and difficulties that might be attendant upon the proposed operation, and the necessity that existed for its immediate performance. She consented, but there was considerable hesitation on the part of her friends and relations; at length they yielded to the exigencies of the case, and resolved, that as it held out the only prospect of relief, and as death was inevitable under existing circumstances, the operation should be performed.

Every thing being now in readiness, and the patient placed in the best position, the operation was begun by grasping the tumor with the left hand, and making the incision with the other; commencing at the upper superior part and extending to its base. The tumor was cut down upon with great care and caution, and after the exposure of the superior surface of the sac, the difficulties were seen to be but beginning. It was then found that this hernia was not one of yesterday's origination, but was of long standing. It was at this point of the operation that the interrogatory was put to her, Have you ever been ruptured before? To which she replied, she had two years before, and that the tumor had always remained.

We discovered that the entire sac was adhering to the surrounding tissue, by strong fibrous bands, which required division with the knife. This circumstance occurring, and unexpectedly, rendered the operation much longer and more tedious than was originally anticipated. Yet, after this general obstruction was removed, so that the finger could be introduced to the point of the stricture at the opening of the external ring, and a division of this tendon effected, together with the bands across the neck of the sac, forming also a part of the stricture, the reduction of the hernia was easily accomplished. It was at first contemplated to return the intestine to the abdomen, and leave the sac to be obliterated; but after it became requisite to destroy this union across the neck of the sac, it was deemed expedient to replace both sac and contents.

The operation developed satisfactorily the cause of our want of success in the application of the taxis — evinced clearly that every effort made to reduce the hernia in this way must have proved ineffectual: because, bound as the sac was by bands of a strong and firm character to the parts adjacent, and constituting also a part of the stricture, it would have been impossible to have returned the intestine to the abdomen, much less both sac and contents. Great apprehensions were entertained lest inflammation might be induced, from the force and violence necessarily exerted to break up this great extent of union; but no unpleasant symptoms manifested themselves, and the patient in a short time recovered, and is now well.

It is true this is an operation of frequent occurrence in the wards of great hospitals and almshouses; yet it is not often we meet with a case under precisely similar circumstances to the one detailed. We know, from the statement of the patient, that this tumor had existed for more than two years, and we cannot suppose that it was made merely by a doubling of the peritoneum constituting the sac, and absolutely of itself forming the tumor; but we must believe that a portion of the bowel was also contained in it — thus showing that a protrusion of intestine through the parietes of the abdomen may exist for an indefinite length of time, and yet not necessarily be impeded in its functions. This woman had become pregnant during the existence of this hernia; proceeded to the full period of utero-gestation, was delivered of a healthy child, and in all this time suffered no inconvenience from it.

The result of this case illustrates clearly the impropriety of allowing a hernial tumor to remain, even though unaccompanied by pain or inconvenience, as it must to a certainty, sooner or later, be the source of much danger and probably death. It speaks the language of reproach to that surgeon or physician whose services may have been called into requisition, and who either made no effort at all to reduce it, or, if any, a slight one; and because the patient was comparatively free from pain, quiets her apprehensions and leaves her to reap the bitter consequences that invariably flow from ignorance and want of skill.

We are gratified to state that Dr. Mount, the operator in this case, is one of our country brethren. From their isolated situation it is not often we find them possessed of the intrepidity or courage to undertake the performance of the more ordinary operations of surgery, much less the accomplishment of one ranking among the boldest and most prominent, requiring the most thorough knowledge of the anatomical structure involved in the operation, with much adroitness in the use of the knife. Although he has been for some years a practitioner of medicine, he is comparatively young as an operative surgeon, yet from his devotedness to his profession, from the zeal with which he pursues the investigation of disease, and the ardent desire he possesses for the acquisition of professional learning, much may be inferred of his future usefulness.

*Cincinnati, June,* 1835.

